# Genetic analysis of the Siberian flying squirrel population in the northern Changbai Mountains, Northeast China: Insights into population status and conservation

**DOI:** 10.1515/biol-2025-1128

**Published:** 2025-07-08

**Authors:** Xinmin Tian, Lanying Shi, Xiaozhen Bai, Ze Wang

**Affiliations:** College of Life Science and Technology, Mudanjiang Normal University, Mudanjiang, 157000, China

**Keywords:** Siberian flying squirrel, cytochrome *b*, microsatellite, population genetic structure, genetic diversity

## Abstract

The Siberian flying squirrel (*Pteromys volans*) is an arboreal, nocturnal, and gliding rodent. It is crucial for maintaining ecosystem balance and the dispersal of forest seeds. In Northeast China, the number of Siberian flying squirrels is decreasing and their habitats are shrinking due to logging and habitat loss. To make more targeted and effective measures to protect and manage the population, there is an urgent need to study the genetic changes in the population, particularly looking at genetic diversity and gene flow. In this study, we collected hair samples from 91 Siberian flying squirrels in a way that did not harm them. Then, we analyzed the DNA in these samples, specifically using cytochrome *b* and microsatellite loci, and examined the genetic diversity and population structure of flying squirrels living in the northern Changbai Mountains of Northeast China. The results indicated that the genetic diversity in the populations was high. However, a high proportion of rare haplotypes and a low frequency of alleles indicated that the genetic diversity might decline in the future. There were significantly low to moderate levels of genetic differentiation among the four populations. According to our STRUCTURE analysis, the four geographical populations belonged to three genetic clusters (Fangzheng, Bin County, and Weihe–Muling). The isolation by distance model could not effectively explain the current pattern of the population genetic structure. The haplotype network showed no clear phylogeographic pattern among the four geographic populations, indicating that the geographic barriers between the flying squirrels might have formed only recently. To better protect the Siberian flying squirrels, conservation methods should be further improved. For example, habitat restoration and ecological corridor construction should be carried out to increase gene exchange and help the population recover and grow faster.

## Graphical Abstract

**Figure:**
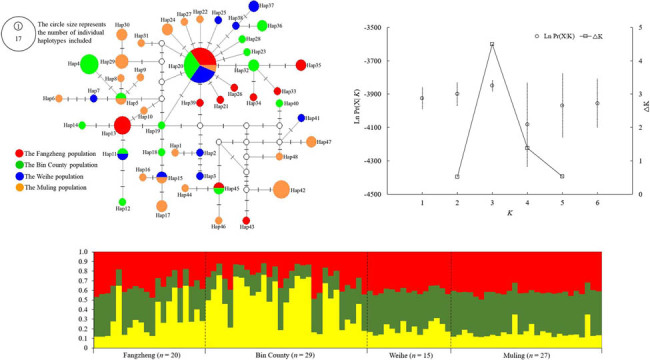

## 1 Introduction

The Siberian flying squirrels (*Pteromys volans*) are nocturnal, tree-dwelling rodents that can glide through the air. They are mostly found in the northern Eurasia, in places like Latvia, Estonia, Finland, Russia, Mongolia, Japan, South Korea, North Korea, and China [1]. The species is under threat to varying degrees in many areas due to habitat loss and fragmentation. The species is on the verge of extinction in Latvia, and is listed as endangered (EN), vulnerable (VU), and near threatened (NT) in South Korea, Estonia, and Finland [2], respectively. In China, the Siberian flying squirrel is the only species belonging to the Pteromys genus of the Sciuridae family. It lives primarily in the northern forests of Northeast China, North China, and Northwest China [1]. As a key indicator species for sustainable forest management [3], the Siberian flying squirrel is of great ecological, scientific, and social value, and has been included in China’s catalogue of terrestrial wildlife. Furthermore, it is classified as VU in China’s Vertebrate Red List [4]. The Siberian flying squirrel reproduces at a low rate, usually bearing one or two litters per year, with 2–4 offspring per litter. Most individuals live only 1–2 years [5], which slows population growth and makes the species even more vulnerable. Globally, numerous studies have been conducted on various aspects of the Siberian flying squirrel, including its morphology, home range, nest-site utilization, habitat selection, genetic diversity, and molecular evolution [6–11]. These studies have provided critical insights to support its protection. In contrast, research in China remains limited. Early studies in China primarily focused on tree-hollow insulation mechanisms, activity rhythm in captivity, molecular marker screening, and taxonomic status of the flying squirrels. Notably, most of these studies have been published in Chinese, which limits their accessibility to the broader scientific community [12–15].

The northeastern region of China, including the Greater Khingan Mountains, Lesser Khingan Mountains, Changbai Mountains, Songnen Plain, and Sanjiang Plain, has historically experienced extensive deforestation and habitat degradation. These environmental pressures have disrupted gene flow among wildlife populations leading to inbreeding, lower genetic diversity, and the accumulation of harmful mutations in small populations. The survival of local wildlife is thus under threat [15–17]. Siberian flying squirrel is a typical tree-dwelling forest species that nests primarily in tree cavities. It is particularly vulnerable to habitat changes [2]. Since modern times, large-scale deforestation, activities that transformed natural landscapes into farmland, and rapid population growth in the Northeast China have all reduced the size of the mature forest habitats, which the Siberian flying squirrel rely on for nesting and foraging. Consequently, the species’ population and distribution are declining [15–17]. To prevent forest degradation, China has implemented a series of effective measures, including the launch of the Natural Forest Protection Program in 2000 and a comprehensive ban on commercial logging of natural forests in 2014 [16]. Research indicates that Siberian flying squirrels prefer to live in trees that are 20–50 years old [2]. Even though there have been efforts of reforestation in the Northeast, it is still hard to find suitable habitats for Siberian flying squirrels. According to field surveys, Siberian flying squirrels are not as active in many forests as they used to be. Their populations are shrinking due to habitat loss and fragmentation. As forests are getting cut down or broken into smaller patches, squirrels in one area may be isolated, leading to hindered gene flow and weaker genetic diversity. To better protect the species and its habitats, it is urgent to study how their genetics are responding to these changes. This study uses two genetic tools: mitochondrial DNA (cytochrome *b*) and nuclear DNA (microsatellite markers) to analyze the genetic diversity, the demographic history, and the genetic differentiation of the Siberian flying squirrels in the northern Changbai Mountains. The findings provide valuable insights for scientists to protect the species and its habitats.

## 2 Materials and methods

### 2.1 Study area and sampling

**Figure 1:**
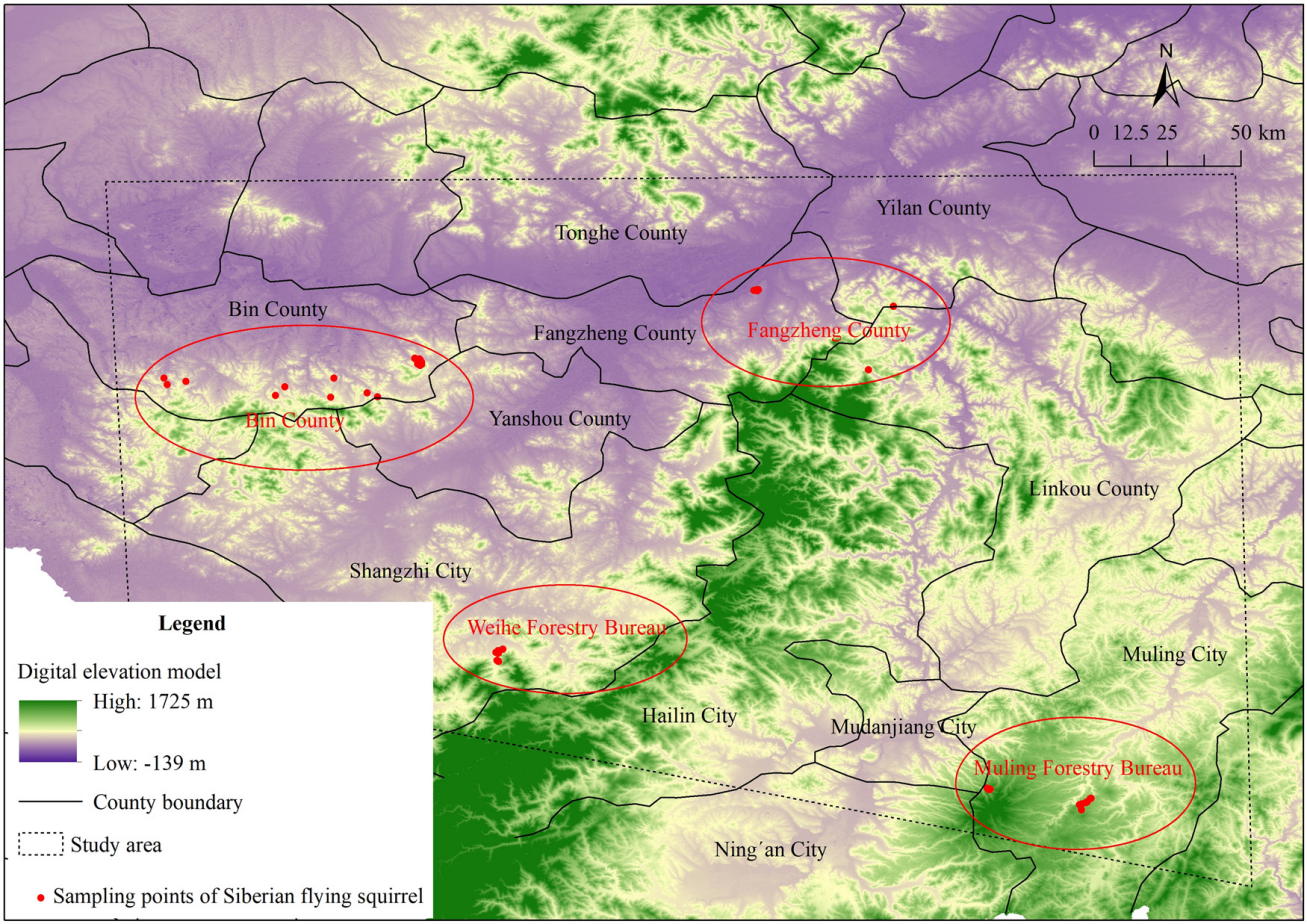
Sampling sites of this study.

During 2022–2024, we conducted field surveys in both autumn and winter in the northern part of the Changbai Mountains, specifically in forests of the Zhangguangcai Mountains and Laoye Mountains. We found four main regions where the Siberian flying squirrels still have notable populations: Bin County, Fangzheng County, Weihe Forestry Bureau, and Muling Forestry Bureau. These four regions together span about 50,000 km^2^ and they are all located in the southern part of Heilongjiang Province, China. Each of these locations is about 110–250 km apart from the others (Figure 1).

In this study, we used nondestructive methods to collect hair samples. When we found a Siberian flying squirrel nest in a tree cavity, we used a net at the entrance to catch the squirrel when it came out. We plucked about 20 hairs from the tail of each Siberian flying squirrel and stored the hairs in sealed bags. GPS coordinates were recorded at the sampling locations. To avoid taking samples from the same squirrel twice, we clipped a small patch of tail hair as a marking. After sampling, we released the squirrels safely back into the wild. A total of 91 hair samples were collected from the four study sites. These samples were stored at −20°C (in a freezer) for further analysis (Table 1).

**Table 1:** Information about collected samples

Study area	Population	Geographic position	Individual number	Coordinate range
Fangzheng County	Fangzheng	Northeastern Zhangguangcai Mountains	20	45°40′–45°55′N, 129°09′–129°34′E
Bin County	Bin County	Northwestern Zhangguangcai Mountains	29	45°30′–46°01′N, 126°55′–128°19′E
Weihe Forestry Bureau	Weihe	Southern Zhangguangcai Mountains	15	44°47′–44°49′N, 128°21′–128°22′E
Muling Forestry Bureau	Muling	Southern Laoye Mountains	27	44°19′–44°24′N, 129°51′–130°11′E


**Ethical approval:** The research related to animal use has been complied with all the relevant national regulations and institutional policies for the care and use of animals, and has been approved by the Experimental Animal Welfare Ethics Committee of Mudanjiang Normal University, 15 September 2022 (Review number: IACUC-MNU-0-901).

### 2.2 DNA extraction and PCR amplification

For genetic analysis, we extracted DNA from 20 hair roots per sample and used a stereoscope to cut off the hair roots and transfer them to a 2.0 mL tube. Using the “TIANamp Micro DNA Kit” (Tiangen, China), we extracted DNA based on the manufacturer’s instructions. To amplify the complete sequence of mtDNA cytochrome *b* gene, we adopted primers CBF: ACATGGAATCTAACCATGACCAA and CBR: AGACTTCATTGTTGGTTTACAAGAC [15]. Eight specific microsatellite loci of the Siberian flying squirrel were studied [14,15] (Table 2). PCR amplification of cytochrome *b* and microsatellite loci was performed using the following conditions: an initial denaturation step at 94°C for 5 min, followed by 35 cycles of strand denaturation at 94°C for 30 s, annealing at 55°C for cytochrome *b* and 50–59°C for microsatellites for 30 s, and elongation at 72°C for 30 s. A final elongation step was carried out at 72°C for 10 min.

**Table 2:** Primer sequence and size range of the eight microsatellite loci (Ta: Annealing temperature)

Primer name	Sequence (5′–3′)	Size range (bp)	Ta (°C)	Fluorophores
Pvol10	F: GTCATAACATCAGTCTTTGG	76–130	50	FAM
	R: ATCACAAAAAAATAAATAAAAGTC			
Pvol41	F: AGGAAATAGGTCTAGTATATGG	108–144	50	ROX
	R: TGGAGTATATAATTTTTCCTG			
PvolE5	F: GCACAATTTCAGCTGCTTACC	139–177	50	HEX
	R: TGAGCTAGGGACTACATGATATGG			
PvolE6	F: TCCTTACTAATGTGAACCCTGACA	161–209	50	TAMRA
	R: CAGTCTTCAAGCACACTTCCT			
Hlep59	F: AATAAATGCTGCTGAAACAAACTC	294–360	59	FAM
	R: GCTGTGCATTAGCCTCAAAG			
Hlep72	F: GCCAAACCACTGCTATCC	194–236	55	ROX
	R: GKGRTAATCCTAGCCACTTG			
Hph17	F: GAGTCCAKKGCCAAAKGAGA	158–186	59	HEX
	R: AGCCTGGAAACTAGGACAGTG			
ScnFO35	F: GATGGACATCTGAAATAGTGAGA	152–180	55	ROX
	R: ACACTGGGCTAAACAACAAA			

Amplifications were performed with a 20 μL reaction volume, containing 2 μL of 10× reaction buffer, 1.6 μL of 2.5 mmol/L dNTPs, 0.5 μL of 10 μmol/L each primer, 0.5 units of Ex Taq DNA polymerase (TaKaRa, Japan), and about 0.1 μg of template DNA. The PCR products were visualized via electrophoresis on a 2% agarose gel. To precisely determine the length of each successfully amplified microsatellite locus, capillary electrophoresis was conducted using the ABI 3730XL sequencer (Applied Biosystems Inc., America). To make sure the obtained genotypes were highly accurate, we used a multi-tube PCR amplification protocol with three positive PCR amplifications per microsatellite locus. In three positive PCR amplifications, two distinct alleles appeared in at least two tests, so it was recorded as heterozygous. Moreover, the same allele appeared in all three tests, so it was recorded as homozygous [15,18]. The paired-end sequencing and genotyping work on all the amplified products were done by Sangon Biotech Co., Ltd (Shanghai, China).

### 2.3 Data analysis

To evaluate population genetic diversity, we used Clustal X 2.1 [19] to align the mitochondrial cytochrome *b* sequences from different individuals. Then we adopted DnaSP 5.10 [20] to calculate the total number of mutation sites, the number of haplotypes, haplotype diversity (*H*_d_), and nucleotide diversity (*P*_i_). During the microsatellite data analysis, we used GenAlEx 6.0 [21] to calculate the number of alleles (*N*_a_) and the number of effective alleles (*N*_e_). We also calculated the observed heterozygosity (*H*_o_) and the expected heterozygosity (*H*_e_) in the populations. Additionally, using the Excel Microsatellite Toolkit v3.1 program (MS Tools) [22], we determined the polymorphic information content (PIC) of each locus and population.

With the microsatellite data, we evaluated the demographic history of populations and used the GenAlEx 6.0 [21] to compute the inbreeding coefficient (*F*_is_) in the population. We also used Genepop 4.0 with statistical tests to see if the real data fits Hardy–Weinberg equilibrium prediction. Deviation from Hardy–Weinberg equilibrium was tested using Fisher’s exact tests with unbiased *p*-values derived by a Markov chain method [23]. To test recent genetic bottlenecks, we should first measure the deviations from expected heterozygosity under the assumption of mutation drift equilibrium. The Bottleneck 1.2 software was adopted in this process [24]. When choosing between the stepwise mutation model and the two-phase model (TPM), we found that TPM was the most suitable model for microsatellite analysis [25]. The data were then analyzed with the recommended settings [24].

To assess the structure of the population, we analyzed cytochrome *b* sequences. Arlequin 3.11 [26] was used to calculate the genetic differentiation coefficient (*F*_ST_) and associated *p*-value, gene flow (*N*_m_), and analysis of molecular variance (AMOVA) between populations. A *p*-value less than 0.05 was considered a significant level of genetic differentiation. Network 4.6 [27] was used to build a median-joining network, and analyze the evolutionary relationship between haplotypes. Second, we conducted an in-depth analysis of the population structure using microsatellite data. Population differentiation was assessed using two methods. The first method was using traditional *F*_ST_ to compare the observed averaged allele distributions among *a priori* defined four populations. *F*_ST_, *p*-value, *N*_m_, and AMOVA across the study area and between pairs of sampling sites were calculated using the GenAlEx 6.0 [21]. Although robust population structure analysis can be obtained with *F*_ST_, the *a priori* subdivision of populations can be very subjective and may greatly affect the estimates of population structure [28]. The second method implemented individual-based Bayesian clustering in the program STRUCTURE 2.3.4 [29], in which the number of potential genetic clusters (*K*) was inferred instead of defining populations as *a priori*. The most likely number of populations (*K*) was estimated by conducting 20 independent runs for *K* = 1–6 using a burn-in of 100,000 replications, 10,000 Markov chain Monte Carlo steps. The results from the STRUCTURE program were then uploaded to Structure Harvester Web [30] to analyze and visualize the output. The optimal *K* of population genetic structure was determined based on the maximum value of the Δ*K* and Ln Pr (X|*K*).

To check if genetic differentiation between sampling sites followed the isolation–by-distance (IBD) model, we performed the Mantel test in GenAlEx 6.0 [21]. During the tests, the significance of the regression between pairwise genetic distances, expressed as *F*_ST_/(1 − *F*_ST_), was assessed in relation to the natural log-transformed geographical distance [28]. Statistical significance was determined through 10,000 permutation tests.

## 3 Results

### 3.1 Genetic diversity

A total of 91 mtDNA cytochrome *b* sequences (1,140 bp) were analyzed. According to the results, there were 63 mutations, of which 25 were singleton variable sites and 38 were parsimony informative sites. With an average transition/transversion ratio of 33.5, the majority of base substitutions were transitions. There were no insertions or missing found. Among the 91 cytochrome *b* sequences, 48 distinct haplotypes were identified with 10, 14, 10, and 21 haplotypes found in the populations from Fangzheng, Bin County, Weihe, and Muling, respectively. According to the genetic diversity parameters, the average haplotype diversity (*H*_d_) across the populations was 0.955 (ranging from 0.859 to 0.980) and the average nucleotide diversity (*P*_i_) was 0.509% (ranging from 0.339 to 0.616%) (Table 3).

**Table 3:** Genetic diversity of Siberian flying squirrel populations based on cytochrome *b* gene

Population	Individual number	Haplotype number	Haplotype diversity	Nucleotide diversity (%)
Fangzheng	20	10	0.863	0.339
Bin County	29	14	0.909	0.616
Weihe	15	10	0.859	0.376
Muling	27	21	0.980	0.530
The whole population	91	48	0.955	0.509

According to the microsatellite analysis, two populations – the Fangzheng and Bin County populations deviated significantly from Hardy–Weinberg equilibrium, which suggests a degree of heterozygote deficiency (*p* < 0.05). The inbreeding coefficients (*F*_is_) were positive for the Fangzheng and Bin County populations (*F*_is_ = 0.008 and 0.046, respectively), while negative for the Weihe and Muling populations (*F*_is_ = −0.061 and −0.066, respectively). The overall inbreeding coefficient was negative (*F*_is_ = −0.018). This means inbreeding is occurring in the Fangzheng and Bin County populations (Table 4). Tests for genetic bottleneck showed that the Fangzheng population had a statistically significant bottleneck under the TPM model Wilcoxon test (*p* < 0.05), and the Bin County population was close to critical significance (*p* = 0.057), indicating that both populations had recent genetic bottlenecks.

**Table 4:** Details of the studied microsatellite loci

Population	*N*_a_	*N*_e_	*H*_o_	*H*_e_	PIC	*F*_is_	*P*_HW_
Fangzheng	12.4	8.8	0.869	0.898	0.864	0.008	0.015
Bin County	12.8	8.1	0.832	0.887	0.860	0.046	0.021
Weihe	12.0	8.5	0.925	0.903	0.861	−0.061	0.999
Muling	14.4	8.8	0.921	0.882	0.853	−0.066	0.982
The whole population	12.9	8.6	0.887	0.892	0.891	−0.018	0.269

Mean number of different alleles (*N*_a_), mean number of effective alleles (*N*_e_), polymorphic information content (PIC), observed and expected heterozygosity (*H*_o_, *H*_e_), inbreeding coefficient value (*F*_is_), probability value for Hardy–Weinberg tests (*P*_HW_).

The PIC for the eight microsatellite loci ranged from 0.862 to 0.945, showing a high polymorphism (PIC > 0.5). The PIC values across the four populations ranged from 0.853 to 0.864, with an overall PIC of 0.891. On average, the number of alleles (*N*_a_) was 12.9 (ranging from 12.0 to 14.4), and the effective number of alleles (*N*_e_) was 8.6 (ranging from 8.1 to 8.8). The average effective number of alleles in the four populations was significantly lower than the observed allele number (*p* < 0.01). Across the populations, the average observed heterozygosity (*H*_o_) was 0.887 (ranging from 0.832 to 0.925), and the average unbiased expected heterozygosity (*H*_e_) was 0.892 (ranging from 0.882 to 0.903) (Table 4).

### 3.2 Population structure

**Figure 2:**
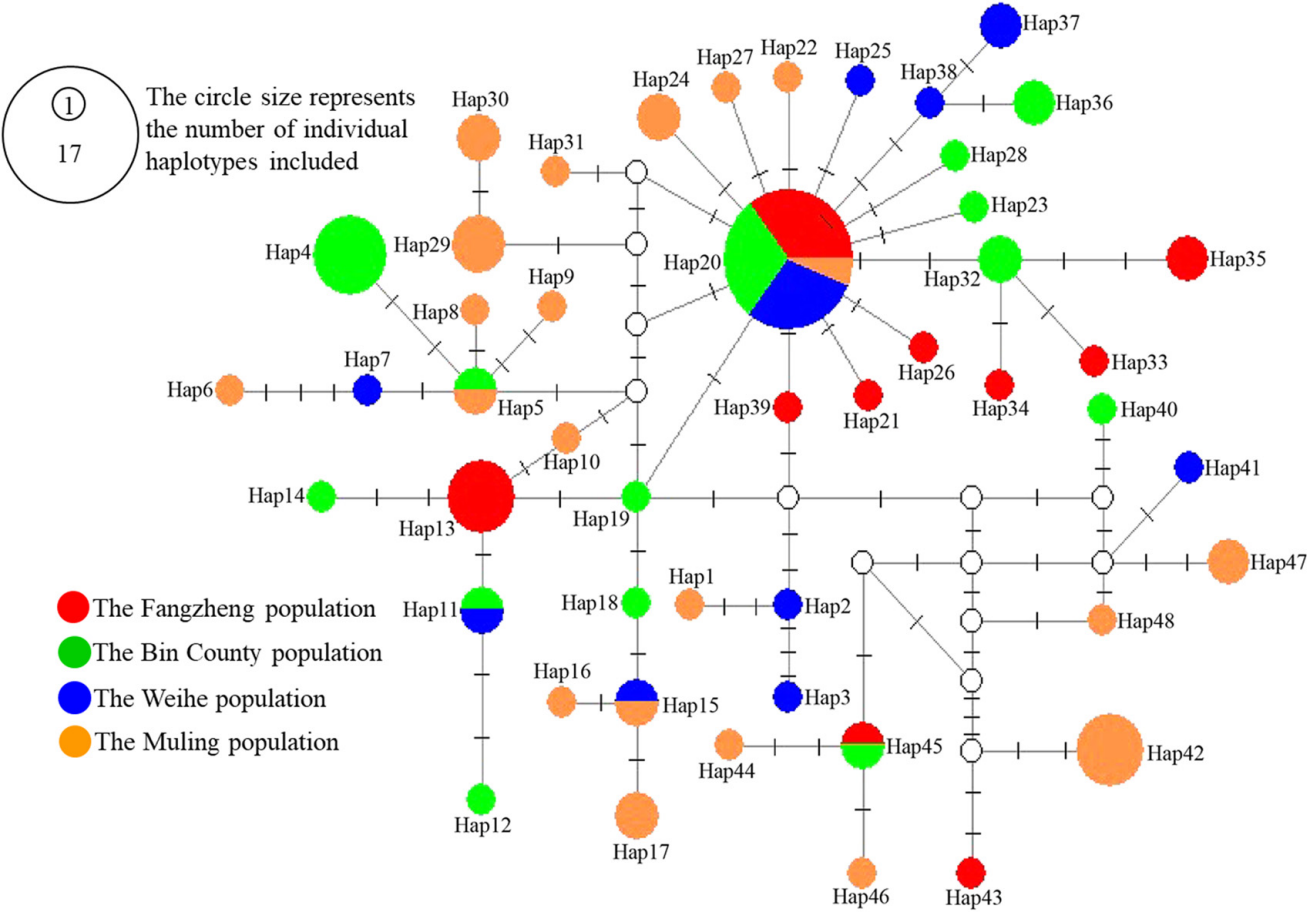
Haplotype network for Siberian flying squirrel based on cytochrome *b* gene. The small hollow circles indicate missing haplotypes, and short bars crossing network branches indicate mutation steps.

The cytochrome *b* analysis showed that the genetic differentiation coefficient (*F*_ST_) among the populations ranged from 0.036 to 0.065. This is a significant genetic differentiation among the four geographic populations of Siberian flying squirrel (*p* < 0.05) (Table 5). The gene flow (*N*_m_) between the populations ranged from 7.18 to 13.22. According to the Mantel test, there was no significant correlation between genetic distance and geographical distance (*r* = −0.164, *p* > 0.05). The haplotype network diagram revealed a mixed distribution of haplotypes across the four geographic populations, without a clear systematic geographic pattern observed. Of the 48 haplotypes, only five (Hap5, Hap11, Hap15, Hap20, and Hap45) were shared between two or more populations. The genetic variation was not evenly distributed across the populations, as most of the haplotypes were found only in some specific geographic populations. In other words, 31 rare haplotypes, found in just one sample each, represented 64.58% of the total (Figure 2).

**Table 5:** Pairwise genetic differentiation coefficient (*F*_ST_) (below diagonal) and associated *p* values (above diagonal) between populations based on cytochrome *b* gene

Population	Fangzheng	Bin County	Weihe	Muling
Fangzheng		0.042	0.036	0.009
Bin County	0.046		0.045	0.027
Weihe	0.042	0.051		0.009
Muling	0.065	0.036	0.046	

**Figure 3:**
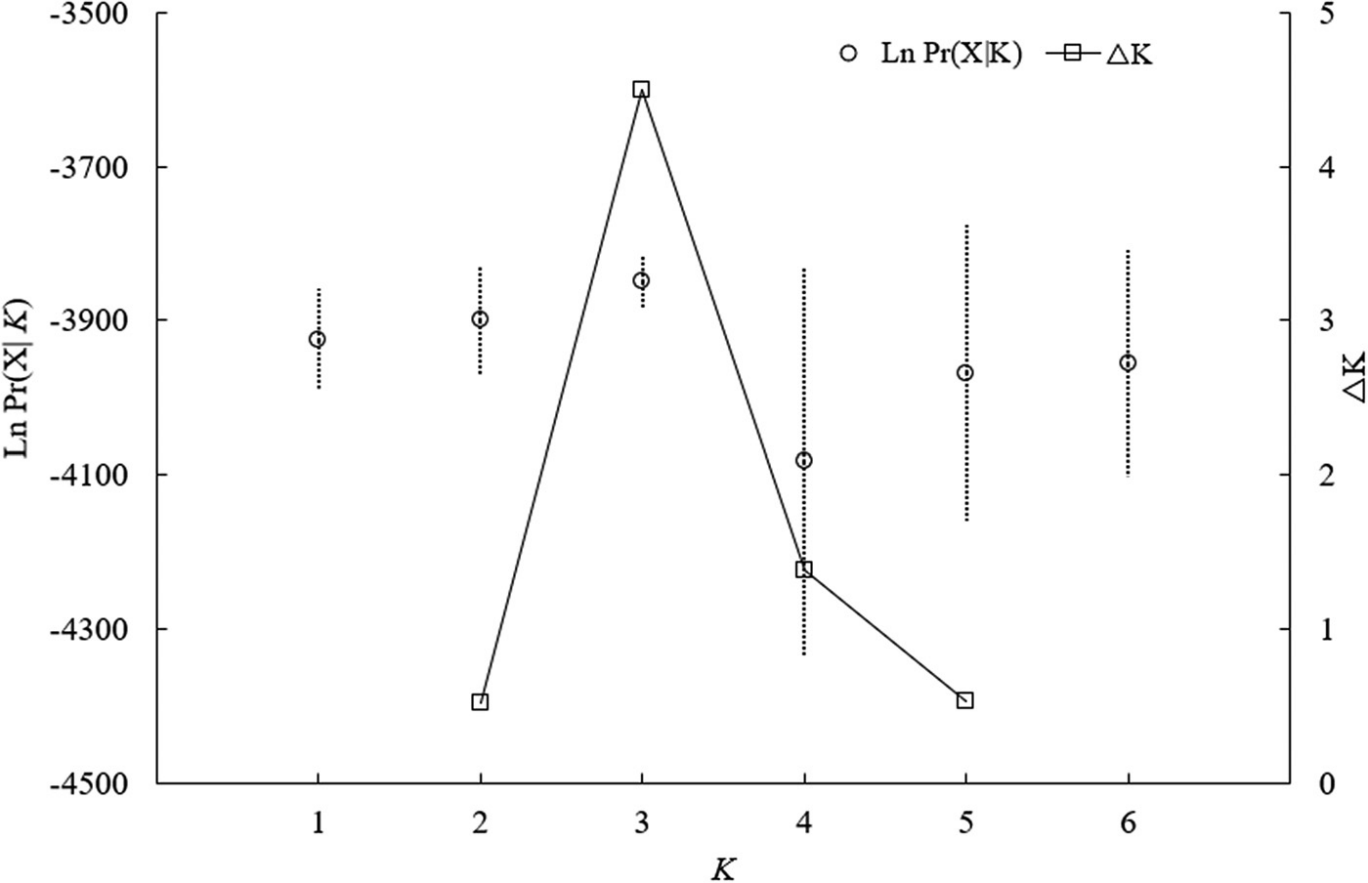
Changing trends of Ln Pr(X|*K*) and Delta *K* from STRUCTURE clustering results for the microsatellite.

**Figure 4:**
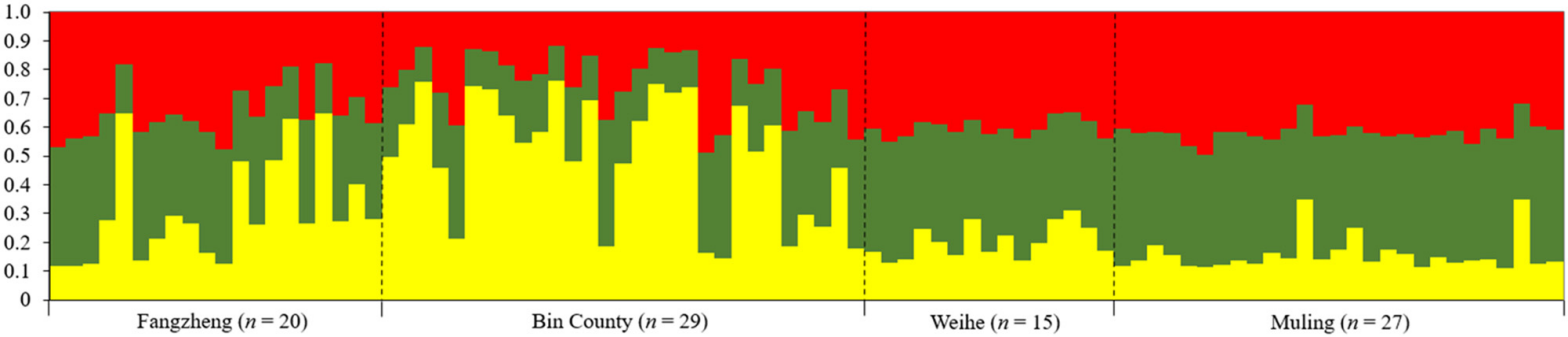
Bayesian clustering result (*K* = 3) of Siberian flying squirrel populations for the microsatellite. Each individual (*n* = 91) is represented by a vertical bar, and the proportions of different colors in the bars are the probability an individual assigned to a certain population. Numbers at the bottom of the graph correspond to the numbers of individual for the sampling estates.

According to the microsatellite analysis, the *F*_ST_ values range from 0.017 to 0.060. Since all values had *p* < 0.05, the genetic differentiation among the four geographic populations were statistically significant (Table 6). The gene flow (*N*_m_) between the populations ranged from 3.92 to 14.46 (Table 7). The Mantel testing showed no significant positive correlation between genetic distance and geographical distance (*r* = 0.751, *p* > 0.05). According to the STRUCTURE analysis, the optimal grouping of the 91 samples was into three genetic clusters (*K*), as indicated by the highest values of Δ*K* and Ln Pr (X|*K*). These clusters matched the locations of Fangzheng, Bin County, and Weihe-Muling. This was closely aligned with the geographic distribution of the samples. However, there was notable gene flow (introgression) among the clusters, particularly between the Fangzheng population and the other two (Figures 3 and 4).

**Table 6:** Pairwise genetic differentiation coefficient (*F*_ST_) (below diagonal) and associated *p* values (above diagonal) between populations based on microsatellite loci

Population	Fangzheng	Bin County	Weihe	Muling
Fangzheng		0.001	0.003	0.001
Bin County	0.039		0.030	0.001
Weihe	0.027	0.017		0.012
Muling	0.060	0.049	0.020	

**Table 7:** Gene flow (*N*_m_) (below diagonal) and geographical distance (km) (above diagonal) between populations based on microsatellite loci

Population	Fangzheng	Bin County	Weihe	Muling
Fangzheng		139.8	133.2	183.6
Bin County	6.16		115.9	253.9
Weihe	9.01	14.46		147.1
Muling	3.92	4.85	12.25	

## 4 Discussion

Since modern times, frequent forest harvesting activities and rapid urbanization in Northeast China have reduced the habitat of Siberian flying squirrel, leading to great changes in its genetic diversity and population structure. Genetic diversity is a fundamental aspect of biodiversity, and understanding its levels, formation, and distribution patterns can help to understand the origin and evolution of this species. It also indicates a species’ ability to adapt to its environment and its potential for future evolution. With our analysis, we can make well-planned and scientifically supported approaches aimed at preserving biodiversity, protecting ecosystems, and ensuring the long-term survival of species [31,32]. Two key metrics in assessing genetic diversity are haplotype diversity (*H*_d_) and nucleotide diversity (*P*_i_). While *H*_d_ measures the variety of distinct haplotypes within a population, *P*_i_ provides a more accurate measure by considering the frequency of these haplotypes. It more accurately reflects the genetic variability within the population [15,33]. In this study, the *P*_i_ for the Siberian flying squirrel populations was found to be 0.509%. This value is lower than that observed in the Russian Primorye population (*P*_i_ = 0.950%) and slightly below the Korean population (*P*_i_ = 0.616%). However, it is significantly higher than the populations in Estonia (*P*_i_ = 0), Finland (*P*_i_ = 0.027%), Karelia in Russia (*P*_i_ = 0.097%), and Hokkaido, Japan (*P*_i_ = 0.219%) [10,34,35]. This indicates that the Siberian flying squirrel populations in the northern Changbai Mountains show a relatively high genetic diversity compared to other regions. The observed values of haplotype diversity (*H*_d_ = 0.955) and nucleotide diversity (*P*_i_ = 0.509%) meet the criteria for populations with high genetic diversity (*H*_d_ ≥ 0.5, *P*_i_ ≥ 0.5%) [36,37]. Furthermore, microsatellite analysis revealed that the average observed heterozygosity (*H*_o_) and expected heterozygosity (*H*_e_) for these populations were 0.887 and 0.892, respectively. These values are notably higher than those reported for populations in Finland (*H*_o_ = 0.410–0.772, *H*_e_ = 0.430–0.748) and Estonia (*H*_o_ = 0.608, *H*_e_ = 0.764) [10,34,38]. These results indicate that the Siberian flying squirrel populations in the northern Changbai Mountains have a high genetic diversity, a strong capacity to adapt to environmental changes, and a good evolutionary potential. The analysis provides a solid foundation for future efforts to preserve the species’ resilience and long-term survival.

The study suggests that populations from refugia during glacial periods tend to have higher genetic diversity compared to populations in other areas [39,40]. The Siberian flying squirrel population observed in this study shows notably higher genetic diversity than foreign populations. This implies that the Changbai Mountain may have provided sanctuary for these squirrels when much of their typical forest habitat was rendered uninhabitable during the ice age. Based on the analysis of the cytochrome *b* gene, we have identified three distinct genetic lineages of Siberian flying squirrel: the Far Eastern lineage, Northern Eurasian lineage, and Hokkaido (Japan) lineage. Populations from regions belonging to the Far Eastern lineage, such as Heilongjiang in China, Korea, and the Russian Primorye, all display high levels of genetic diversity than other lineages [15,41]. Multiple refugia for Siberian flying squirrel are proposed to have existed across Eurasia during the Quaternary glaciations, with these populations then expanding over northern Eurasia following the ice age, hence influencing the geographic distribution of lineages of present days. Arboreal species, which rely on forested environments for shelter and food, would have been confined to the remaining forested areas during the Pleistocene. Thus, the refugia of arboreal species like the Siberian flying squirrel may have been more dependent on forest dynamics than terrestrial rodents [41]. According to phylogeographic studies, during the Last Glacial Maximum, certain regions in Northeast Asia, specifically the Changbai Mountains, the Korean Peninsula, and the Russian Far East, may have mixed coniferous-broadleaf forests, which provided stable refugia [42]. This further supports our observation of high genetic diversity in the populations of the Far Eastern lineage. Despite the high genetic diversity of the population studied, a high proportion of rare haplotypes in the cytochrome *b* gene (31/48) was also detected. In addition, the number of effective alleles at microsatellite loci was significantly lower than the actual number of alleles (*p* < 0.01). This indicates a risk of haplotype and allele loss in future population development. The high proportion of rare haplotypes and alleles in the population may be a sign of genetic bottlenecks and inbreeding, where the flying squirrel population is drastically reduced. This hypothesis is further confirmed by genetic bottlenecks and inbreeding observed in the Fangzheng and Bin County populations. Therefore, it is important to further protect and manage Siberian flying squirrel populations in Northeast China against the decrease in genetic diversity.

Genetic differentiation and gene flow are crucial indicators for evaluating the genetic structure of populations [43]. The genetic differentiation coefficient, *F*_ST_, is used to assess the genetic divergence between populations. *F*_ST_ ≤ 0.05 indicates low genetic differentiation; 0.05 < *F*_ST_ ≤ 0.15 reflects moderate genetic differentiation; and *F*_ST_ > 0.15 represents great genetic differentiation between populations [44]. In this study, the *F*_ST_ values from two molecular markers reveal significant, low, or moderate levels of genetic differentiation among the four geographical populations of Siberian flying squirrel. Mantel tests failed to detect the correlation between genetic and geographical distances. This suggests that the IBD model does not fully explain the current genetic structure of the populations, so we need to evaluate isolation-by-environment factors. The species’ dispersal ability and the landscape configuration are among the most important factors that influence population genetic structure [15,28,37]. Although Siberian flying squirrel is a gliding species, it has a relatively weak dispersal ability for its small size. Thus, it is more susceptible to the effects of environmental isolation [7,34]. According to the STRUCTURE results, the populations of Weihe and Muling in the southern part of the study area form a distinct genetic cluster. This suggests that there is frequent gene flow between these two populations. Although a portion of the Ning’an farmland is located between the Weihe and Muling populations, continuous forest on both the northern and southern sides of this region act as natural corridors that facilitate the movement of individuals between the two populations. In contrast, the presence of agricultural land corridors in the northern regions, especially between Fangzheng and Shangzhi, along with extensive road networks acts as barriers that limit the movement of the Siberian flying squirrel populations. Studies have also shown that habitat fragmentation is more severe in the northern regions [45], which further restricts individual dispersal and gene flow between the northern populations and those in the south (Figure 1).

There is a noticeable genetic differentiation among populations of the Siberian flying squirrel, but the gene flow (*N*_m_) values between populations all exceed 1.0, and the haplotype network shows a mixed distribution of haplotypes. This suggests that these groups of flying squirrels have only recently become isolated. There has not been sufficient time for the populations to form a monophyletic group. Similar genetic patterns have been observed among populations in Estonia, Russian Karelia, and Finland, which can be attributed to the widespread deforestation that has taken place in these areas [34]. As a typical arboreal forest species that primarily nests in tree cavities, the Siberian flying squirrel populations are highly susceptible to habitat changes [2]. However, the mature forest habitats that they rely on for nesting and foraging have quickly shrunk in recent years, due to the rapid growth in population, logging, agriculture, road construction, and urban development in Northeast China. Field surveys have revealed that the Siberian flying squirrel’s activity is now difficult to detect in many forested areas [15]. The significant genetic differentiation, isolation among populations, and impeded gene flow can lead to a decline in genetic diversity. Thus, we need to carry out fundamental ecological research on Siberian flying squirrel to analyze its population size, distribution patterns, habitat preferences, and evaluations of their status. Moreover, landscape genetics approaches are also essential to identify the environmental factors that affect genetic differentiation. To enhance population growth and genetic exchange of the Siberian flying squirrel, conservation efforts should prioritize the development of landscape-friendly forest areas. This involves integrating natural habitat restoration with targeted artificial interventions such as installing nest boxes, creating crossing structures, and implementing rewilding programs [46,47]. Additionally, improving habitat suitability and connectivity is essential to prevent further decline in genetic diversity and fitness resulting from genetic differentiation.

## 5 Conclusion

The populations of the Siberian flying squirrel located in the Changbai Mountains show a high genetic diversity, indicating that the Changbai Mountains may have served as a glacial refugium during the last ice age. However, due to the decline and fragmentation of suitable habitats, the populations have also experienced severe genetic differentiation, a high proportion of rare haplotypes and alleles, genetic bottlenecks, and inbreeding. The IBD alone may not adequately account for the limited gene flow between populations. Therefore, future research should further assess isolation by environment as a complementary approach. To enhance gene exchange among individuals and accelerate population recovery, it is essential to restore the habitats of the Siberian flying squirrel and build more ecological corridors.
